# Angular dependence correction of MatriXX and its application to composite dose verification

**DOI:** 10.1120/jacmp.v13i5.3856

**Published:** 2012-09-06

**Authors:** Yoshinobu Shimohigashi, Fujio Araki, Hirofumi Tominaga, Junichi Sakata, Keiichi Kawasaki, Nagisa Kanetake, Yuki Iwashita, Saori Yoshimura, Tamami Kawakami, Terunobu Ishihara, Tomoko Okuda, Kasei Kogo

**Affiliations:** ^1^ Kumamoto Radiosurgery Clinic Kumamoto Japan; ^2^ Graduate School of Health Sciences Kumamoto University Kumamoto Japan

**Keywords:** 2D ionization chamber array, angular dependence correction, IMRT, VMAT, composite dose verification

## Abstract

We measured the angular dependence of central and off‐axis detectors in a 2D ionization chamber array, MatriXX, and applied correction factors (CFs) to improve the accuracy of composite dose verification of IMRT and VMAT. The MatriXX doses were measured with a 10° step for gantry angles (θ) of 0°–180°, and a 1° step for lateral angles of 90°–110° in a phantom, with a $30\times 10\;{\text{cm}}^{2}$ field for 6 MV and 10 MV photons. The MatriXX doses were also calculated under the same conditions by the Monte Carlo (MC) algorithm. The CFs for the angular dependence of MatriXX were obtained as a function of θ from the ratios of MatriXX‐measured doses to MC‐calculated doses, and normalized at $\theta =0^{{}^{\circ}}$. The corrected MatriXX were validated with different fields, various simple plans, and clinical treatment plans. The dose distributions were compared with those of MC calculations and film. The absolute doses were also compared with ionization chamber and MC‐calculated doses. The angular dependence of MatriXX showed over‐responses of up to 6% and 4% at θ=90° and under‐responses of up to 15% and 11% at 92°, and 8% and 5% at 180° for 6 MV and 10 MV photons, respectively. At 92°, the CFs for the off‐axis detectors were larger by up to 7% and 6% than those for the central detectors for 6 MV and 10 MV photons, respectively, and were within 2.5% at other gantry angles. For simple plans, MatriXX doses with angular correction were within 2% of those measured with the ionization chamber at the central axis and off‐axis. For clinical treatment plans, MatriXX with angular correction agreed well with dose distributions calculated by the treatment planning system (TPS) for gamma evaluation at 3% and 3 mm. The angular dependence corrections of MatriXX were useful in improving the measurement accuracy of composite dose verification of IMRT and VMAT.

PACS number: 87.55.Qr, 87.56.Fc

## I. INTRODUCTION

The intensity‐modulated radiotherapy (IMRT) technique,^(1)^ which provides intensity‐modulated radiation with a fixed gantry angle, dose rate, and dynamic multileaf collimator (DMLC) movement, is used to obtain dose distributions that are highly conformal to the target while minimizing the dose to the adjacent healthy tissue. Intensity‐modulated arc therapy has recently been developed; it involves one or more arcs provided by beam modulation with simultaneous changes in the gantry speed, DMLC movement, and dose rate. Further, a radiotherapy technique termed volumetric‐modulated arc therapy (VMAT) has been newly developed.^(2)^ Some studies^3^, ^4^ have compared VMAT with IMRT in relation to target coverage, healthy tissue sparing, and treatment time. RapidArc (Varian Medical Systems, Palo Alto, CA) is a technology commercialized as a derivative of VMAT treatment planning and delivery. Ling et al.^(5)^ showed that the DMLC movement, variable dose rate, and gantry speed can be precisely controlled using RapidArc.

The dose delivery for an advanced radiotherapy technique such as IMRT or VMAT must be verified before clinical implementation in order to ensure that the treatment plan can be executed accurately.^(6)^ The dose verification method involves the comparison of the dose distribution calculated by the treatment planning system (TPS) in a phantom with dose distribution measured with a film,^7^, ^9^ or by two‐dimensional (2D) arrays or in an ionization chamber.^(10)^ Although film dosimetry has very good spatial resolution, it requires careful calibration and real‐time measurements are unavailable. It does, however, remain the gold standard for 2D dose verification.

From the perspective of efficient and reliable quality assurance (QA), 2D arrays with ionization chambers or diodes have been widely used and characterized for dose verification of IMRT^11^, ^19^ and VMAT.^20^, ^29^ One such device is the 2D ionization chamber array I'mRT MatriXX (IBA Dosimetry, Bartlett, TN). Amerio et al.^(13)^ described the design and construction of I'mRT MatriXX, whereas Herzen et al.^(17)^ extensively evaluated its dosimetric properties. The clinical application of I'mRT MatriXX has been reported in numerous studies.^13^, ^15^, ^17^, ^19^ I'mRT MatriXX is designed to measure doses for beams that are vertical to its front surface; hence, it cannot be used for composite dose verification with multiple gantry angles. The angular dependence of I'mRT MatriXX would affect the measurement accuracy in composite dose verification of IMRT or VMAT. Dobler et al.^(26)^ investigated the applicability of I'mRT MatriXX to oblique beam incidence and to the composite dose verification of IMRT with multiple gantry angles. They concluded that I'mRT MatriXX could be used for composite dose verification within a dose tolerance of 3% and a distance‐to‐agreement (DTA) of 3 mm, with a relaxed dose tolerance of 4% in the low‐dose region outside the MLC. However, they reported that caution should be taken if the main contribution is irradiated through backscatter material and metal screws.

Recently, attempts have been made to apply a 2D array system to the composite dose verification of IMRT and VMAT. Van Esch et al.^(20)^ considered the angular dependence of a 2D ionization chamber array (Seven29, PTW‐Freiburg, Germany) by using a dedicated octagonal phantom (OCTAVIUS, PTW). Jursinic et al.^(24)^ modified a 2D diode array (MapCHECK, Sun Nuclear, Melbourne, FL) by considering the angular dependence. MatriXX^Evolutiion^, which is an upgraded version of I'mRT MatriXX, has been developed for composite dose verification of rotational techniques such as VMAT. The angular dependence of ${\text{MatriXX}}^{\text{Evolution}}$ was improved by replacing the metal screws on the body with plastic screws and adding a scatterer under the detectors. Wolfsberger et al.^(23)^ conducted a comprehensive study on the angular dependence of ${\text{MatriXX}}^{\text{Evolution}}$ and its correction method. However, they assumed that the angular correction factor (CF) is constant within the detector plane. Recently, Boggula et al.^(30)^ investigated the angular dependence of all the detectors of MatriXX and used its CFs for the composite dose verification of VMAT. However, they have not been investigated for the composite dose verification of IMRT.

With this background, we undertook this study to evaluate the angular dependence of MatriXX detectors, including the off‐axis detectors, and to establish a comprehensive correction method. We then used this method to improve the measurement accuracy for the composite dose verification of IMRT and VMAT.

## II. MATERIALS AND METHODS

All measurements were performed using a Novalis TX system equipped with a high‐definition MLC (Varian Medical Systems; BrainLAB, Feldkirchen, Germany) for 6 MV and 10 MV photon beams. The ionization chamber and film measurements were made using a PTW‐TN31010 Semiflex 0.125 cc thimble chamber and ISP‐RTQA2 radiochromic film, respectively. The optical density of RTQA2 was converted into an absolute dose measured with the ionization chamber. The dose distributions for $10\times 10{\;\text{cm}}^{2}$, $15\times 15{\;\text{cm}}^{2}$, and $20\times 20{\;\text{cm}}^{2}$ fields, various simple plans, and IMRT plans were calculated using the Monte Carlo (MC) dose algorithm installed in the iPlan RT (Version 4.1.2, BrainLAB).^31^, ^33^ MC calculations were performed via a full MLC geometry simulation with a spatial resolution of 2 mm and variance of 1%. The dose distributions for VMAT (RapidArc) plans were calculated using the anisotropic analytical algorithm (AAA) installed on Eclipse (Version 8.6, Varian). The dose distributions for both TPSs were calculated by considering attenuation by the treatment couch.^34^, ^35^ In the iPlan RT, the virtual couch top was inserted in the CT (computed tomography) datasets; meanwhile Hounsfield Unit values were assigned to the couch model in Eclipse, as described by Vanetti et al.^(34)^ Both the couch models agreed within 1.5% with measured dose by using the ionization chamber. The calculated dose distributions were exported to OmniPro‐I'mRT (Version 1.7, IBA Dosimetry), which is the analysis software installed in the ${\text{MatriXX}}^{\text{Evolution}}$ system. The dose distributions of RTQA2 and MatriXX were compared and analyzed with those of the TPS by the gamma evaluation method.^36^, ^37^ The MatriXX and TPS dose distributions were compared with the absolute dose mode. The calculated and measured dose distributions were linearly interpolated to a pixel size of 1 by 1 mm.^37^, ^39^ The gamma evaluation was conducted with dose tolerances of 2% and a DTA of 2 mm, dose tolerances of 3% and a DTA of 3 mm, with a 5% threshold to exclude the low‐dose region.

### A. MatriXXEvolution system

**Figure 1:**
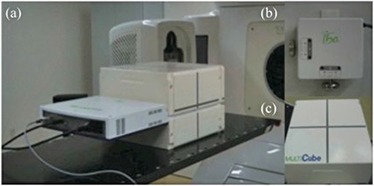
Combination of MatriXX and MULTICube Lite phantom: (a) Orientation normally used for the dose verification of IMRT and VMAT; (b) gantry angle sensor; (c) MULTICube Lite phantom.

The ${\text{MatriXX}}^{\text{Evolution}}$ system consists of a MatriXX device, a MULTICube Lite (IBA Dosimetry) phantom, a gantry angle sensor, and OmniPro‐I'mRT software, as shown in Fig. 1. The design and construction of MatriXX were reported by Amerio et al.^(13)^ The dose distribution of MatriXX was measured in the movie mode with a sampling rate of 200 ms/snap. This high sampling rate was used to resolve gantry angle differences for VMAT plans with arc segments and to confirm the behavior of dynamic MLC during the radiation for IMRT plans. (Figure 1a) shows the MatriXX device inserted into the MULTICube Lite phantom. The MULTICube Lite was made of Plastic Water and was 31.4 cm long, 22 cm high, and 34 cm wide.^38^, ^39^ The slab phantom that set RTQA2 film or the PTW‐TN31010 chamber was also inserted into the MULTICube Lite phantom to compare with MatriXX dose distributions at the same position.

The MatriXX‐measured doses were averaged over $1\times 1{\;\text{cm}}^{2}$ regions of interest (ROIs) that cover the sensitive volume of the PTW‐TN31010 chamber. The gantry angle sensor was used for online measurement with MatriXX at an arbitrary gantry angle.

### B. Angular dependence of MatriXX and its correction factors

**Figure 2:**
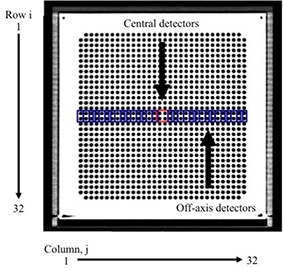
Regions of interest (ROIs) corresponding to the sizes and locations of MatriXX detectors (column: j=1, 2, ..., 32; row: i=16, 17) were configured in the CT images. The doses of ROIs were calculated with the MC algorithm on iPlan. The dose of the central detectors was defined as the average dose of four detectors at the center (red line). In addition, the dose of the off‐axis detectors was defined as the average dose of two detectors (row: i=16, 17) at column j=1,2,...,32 (blue line).

CT images of MatriXX inserted into MULTICube Lite were taken by a LightSpeed RT 16 CT scanner (GE Healthcare, Waukesha, WI) and exported to iPlan. As shown in Fig. 2, ROIs corresponding to the sizes and locations of the MatriXX detectors (column: j=1, 2, ..., 32; row: i=16, 17) were configured in the CT images. The dose of the central detectors was defined as the average dose of four detectors at the center (red line). The dose of the off‐axis detectors was determined with the average dose of two detectors (row: i=16, 17) at column j=1,2,...,32 (blue line). The doses of the ROIs were calculated with the MC algorithm on iPlan. The angular dependence of MatriXX was measured with a 10° step for gantry angles of 0°–180° and a 1° step for lateral angles of 90°–110° at a $30\times 10{\;\text{cm}}^{2}$ field for both 6 MV and 10 MV photons. For gantry angles of 180°–360°, we assumed that the angular dependence of MatriXX detectors was similar to that for gantry angles of 0°–180° because the setup accuracy was improved by using the MULTICube Lite. MatriXX was vertically set up to avoid irradiation through the treatment couch.

CFs were calculated from the ratio of the MatriXX‐measured dose to the MC‐calculated dose as a function of gantry angle θ. ${\text{CF}}_{\text{ij}}$ (θ) of the MatriXX detector at row i and column j at θ was defined as:

(1) $${\text{CF}}_{\text{ij}}(\theta )=N_{\text{ij}}\frac{D_{\text{ij}}^{\text{MatriXX}}(\theta )}{D_{\text{ij}}^{\text{MC}}(\theta )}$$

where *D* ^*MatriXX*^ *(*θ*)* and *D* ^*MC*^ *(*θ*)* are the MatriXX‐measured and MC‐calculated doses at θ, respectively, and $N_{\text{ij}}$ is a factor normalized at θ=0°.^38^, ^39^ In addition, differences in CFs between the central and off‐axis detectors at gantry angles were calculated as follows:

(2) Difference (%)=off‐axis detector's CF(θ)‐central detector's CF(θ)central detector's CF(θ)×100

### C. Correction methods for angular dependence and their validations

Two correction methods were used for the angular dependence of MatriXX. The first method, which is based on “central correction,” applies the CF of the central detectors of all MatriXX detectors. The other, based on “entire correction,” applies the CFs of off‐axis detectors (column: j=1, 2, ..., 32; row: i=16, 17) at MatriXX detectors of row i=1,2,...,32 (Fig. 2).

The two correction methods were tested at θ=0°–180° and fields of $10\times 10{\;\text{cm}}^{2}$, $15\times 15{\;\text{cm}}^{2}$, and $20\times 20{\;\text{cm}}^{2}$ for both 6 MV and 10 MV photons. The corrected MatriXX $\left(D_{\text{Corr}}^{\text{MatriXX}}\right)$ doses for the angular dependence were obtained according to the following equation in OmniPro‐I'mRT:^38^, ^39^

(3) $$D_{\text{Corr}}^{\text{MatriXX}}(\theta )=\frac{D_{\text{ij}}^{\text{MatriXX}}(\theta )}{{\text{CF}}_{\text{ij}}(\theta )}$$

The uncorrected, centrally corrected, and entirely corrected MatriXX doses were compared with the doses measured with a PTW‐TN31010 chamber for the central and off‐axis detectors. The dose distributions for each field were compared with those obtained by MC calculations with the gamma evaluation.

### D. Dose verification of various simple plans and IMRT or VMAT plans

The dose distributions of TPS for various simple plans and IMRT or VMAT plans were compared with those measured using MatriXX, a PTW‐TN31010 chamber, and RTQA. The parameters of the simple plans and IMRT or VMAT plans are listed in Tables 1 and 2, respectively. The IMRT plans were performed with fixed gantry angles of 5 to 7 for 6 MV and 10 MV photons. The VMAT plans were three arcs with collimator angles of 45°, 315°, and 90° for 6 MV photons. The three‐arc VMAT plans were used to reduce tongue and groove effects and to resolve “island blocking problem” for the brain treatment site (multiple brain metastases) addressed by Kang et al.^(40)^

**Table 1:** Parameters of simple plans: static and arc delivery with various gantry angles for 6 MV and 10 MV photons. The field sizes were $10\;\times \;10\;{\text{cm}}^{2}$, $15\;\times \;15\;{\text{cm}}^{2}$, and $20\;\times \;20\;{\text{cm}}^{2}$.

*Plan No*.	*Delivery*	*Field Size (cm* ^*2*^ *)*	*Gantry Angle, θ (degrees)*
1	Static	10×10, 15×15, 20×20	0°, 180°
2	Static	10×10, 15×15, 20×20	90°, 270°
3	Static	10×10, 15×15, 20×20	0°, 90°, 180°, 270°
4	Static	10×10, 15×15, 20×20	60°, 120°, 240°, 300°
5	Static	10×10, 15×15, 20×20	45°, 90°, 135°, 225°, 270°, 315°
6	Arc	10×10, 15×15, 20×20	185° to 175°

**Table 2:** Parameters of IMRT and VMAT plans calculated with iPlan MC and Eclipse AAA, respectively.

*Plan No*.	*Energy*	*Delivery*	*Treatment Site*	*Gantry Angle, θ (degrees)*
1	6 MV	VMAT	Brain	3 arc (200° to 160°, 160° to 200°, 200° to 160°)
2	6 MV	VMAT	Brain	3 arc (200° to 160°, 160° to 200°, 200° to 160°)
3	6 MV	IMRT	Neck	210°, 245°, 340°, 10°, 45°
4	6 MV	IMRT	Neck	210°, 240°, 295°, 10°, 180°
5	10 MV	IMRT	Pelvis	205°, 235°, 280°, 80°, 125°, 155°
6	10 MV	IMRT	Prostate	230°, 290°, 330°, 30°, 70°, 130°, 180°

## III. RESULTS

### A. Angular dependence of MatriXX including off‐axis detectors

**Figure 3:**
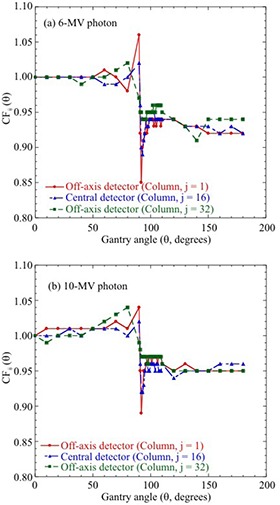
Angular dependence of MatriXX detectors. ${\text{CF}}_{\text{ij}}$ (θ) for the detectors (column: j=1, 16, 32; row: i=16,17) is shown as a function of gantry angle for (a) 6 MV and (b) 10 MV photons. Although not shown here, ${\text{CF}}_{\text{ij}}$ (θ) for other detectors is between off‐axis detectors of columns j=1 and j=32.

Figure 3 shows the angular dependence of MatriXX for 6 MV and 10 MV photons. The angular dependence of MatriXX detectors along rows i=16 and i=17 revealed over‐responses of 6% and 4% at $\theta =90^{{}^{\circ}}$ and under‐responses of 15% and 11% at 92°, as well as 8% and 5% at 180° for 6 MV and 10 MV photons, respectively. The magnitudes of the angular dependence were different for the central and off‐axis detectors, especially around $\theta =90^{{}^{\circ}}$ where the beam is parallel to the detector plane. CFs for other off‐axis detectors were between columns j=1 and j=32. Differences in CFs between the central and off‐axis detectors for different gantry angles are summarized in Table 3. The differences were up to 7% and 6% at around $\theta =90^{{}^{\circ}}$, up to 2.2% and 2.4% at $\theta =0^{{}^{\circ}}-70^{{}^{\circ}}$, and up to 2.0% and 1.9% at $\theta =120^{{}^{\circ}}-180^{{}^{\circ}}$, for 6 MV and 10 MV photons, respectively.

**Table 3:** Differences in correction factors between central and off‐axis detectors for various gantry angles. Difference (%)=((off−axis detector's CF(θ)−central detector's CF(θ))/central detector's CF(θ))×100.

*Photon Energy*	*6 MV*		*10 MV*	
*Gantry angle, θ (degrees)*	*Average*	*Range*	*Average*	*Range*
0°–70°	0.4	−1.0 to 2.2	0.6	−1.0 to 2.4
80°–110°	1.1	−6.1 to 7.0	1	−3.7 to 6.0
120°–180°	0.1	−2.1 to 2.0	0.6	−1.3 to 1.9

### B. Correction methods for angular dependence and their validations

**Figure 4:**
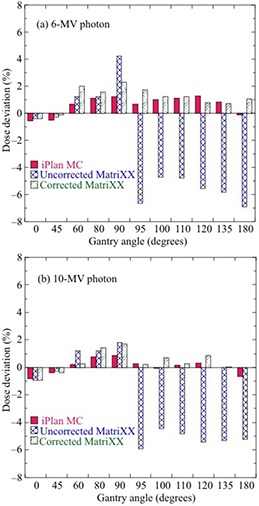
Deviations of iPlan MC‐calculated doses and MatriXX‐measured doses without and with angular correction from PTW‐TN31010 doses at θ=0°–180° for the central axis of a $10\times 10{\;\text{cm}}^{2}$ field for (a) 6 MV and (b) 10 MV photons.

**Figure 5:**
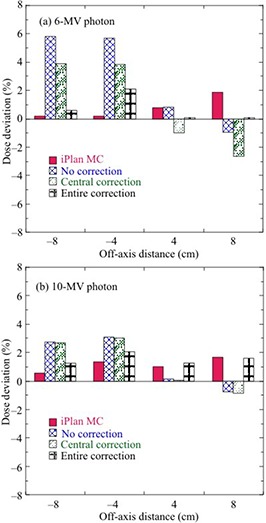
Deviations of iPlan MC‐calculated doses and MatriXX‐measured doses without and with “central correction” or “entire correction” from PTW‐TN31010 doses at θ=90° as a function of off‐axis distance in a $10\times 10{\;\text{cm}}^{2}$ field for (a) 6 MV and (b) 10 MV photons.

Figures 4 and 5 show the deviations of iPlan MC‐calculated doses and MatriXX‐measured doses without and with angular correction, respectively, from the PTW‐TN31010 doses at θ=0°–180° for a $10\times 10{\;\text{cm}}^{2}$ field. In Fig. 4, the MC‐calculated doses were within 1.5% of the PTW‐TN31010 doses for 6 MV and 10 MV photons. The uncorrected MatriXX doses deviated by up to ‐7% and ‐6% for 6 MV and 10 MV photons, respectively. In contrast, the corrected MatriXX doses were within 2% of the PTW‐TN31010 doses for 6 MV and 10 MV photons. For off‐axis distances from −8 cm to +8 cm at θ=90° in Fig. 5, MatriXX doses with “central correction” were within 4% and 3% of the PTW‐TN31010 doses for the 6 MV and 10 MV photons, respectively. Meanwhile, MatriXX doses with “entire correction” were within 2% of the PTW‐TN31010 doses for 6 MV and 10 MV photons.

**Figure 6:**
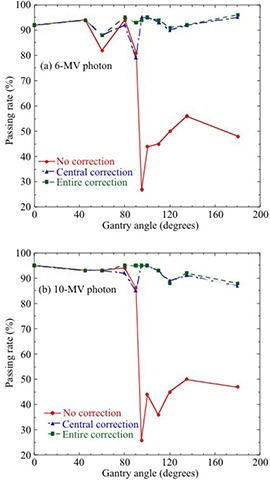
Passing rates between iPlan MC‐calculated and MatriXX dose distributions without and with “central correction” or “entire correction” at θ=0°–180° with a $10\times 10{\;\text{cm}}^{2}$ field for (a) 6 MV and (b) 10 MV photons. Gamma evaluation was performed with 2% dose difference and 2 mm distance to agreement.

**Figure 7:**
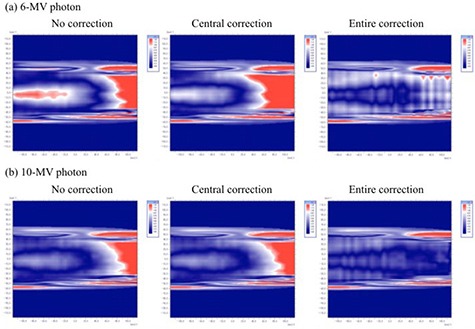
Gamma evaluation analyzed at θ=90° in which was performed with 2% dose difference and 2 mm distance to agreement by using OmniPro‐I'mRT.

Figure 6 shows the passing rates between iPlan MC‐calculated and MatriXX‐measured dose distributions without and with “central correction” or “entire correction” at θ=0°–180° for a $10\times 10{\;\text{cm}}^{2}$ field. The passing rates for “no correction” at gantry angles greater than 90° were less than 56% and 50% for 6 MV and 10 MV photons, respectively. For a 6 MV photon, the passing rates for “no correction,” “central correction,” and “entire correction” at $\theta =90^{{}^{\circ}}$ were 81%, 79%, and 93%, respectively, at 2% dose tolerance and 2 mm DTA. Similarly, the corresponding rates for a 10 MV photon were 86%, 85%, and 95%, respectively. The passing rates for “central correction” decreases 2% and 1% compared to those for “no correction” for 6 MV and 10 MV photons, respectively. In contrast, the passing rates for “entire correction” at gantry angles around 90° were far superior to those for “central correction.” Average passing rates between the iPlan MC‐calculated and MatriXX dose distributions without and with “central correction” or “entire correction” at θ=0°–180° for the $10\times 10{\;\text{cm}}^{2}$, $15\times 15{\;\text{cm}}^{2}$, and $20\times 20{\;\text{cm}}^{2}$ fields are shown in Table 4. The passing rates with “entire correction” were far superior to those for “no correction” and “central correction.” The improvement of the passing rate was remarkable with increasing the field size. Figure 7 shows the gamma evaluation analyzed at θ=90° in Fig. 6, which was performed with 2% dose tolerance and 2 mm DTA using OmniPro‐I'mRT. “Entire correction” is significantly better than “no correction” and “central correction.”

**Table 4:** Comparison in average passing rates (%) between iPlan MC‐calculated and MatriXX dose distributions without and with “central correction” or “entire correction” at θ=0°–180° for $10\;\times \;10\;{\text{cm}}^{2}$, $15\;\times \;15\;{\text{cm}}^{2}$, and $20\times 20\;{\text{cm}}^{2}$ fields with (a) 6 MV and (b) 10 MV photons. Gamma evaluation was performed with 2% dose difference and 2 mm distance‐to‐agreement (DTA) criteria.

(a).			
	*Angular Correction*		
*Field Size (cm* ^*2*^ *)*	*No Correction*	*Central Correction*	*Entire Correction*
10×10	64.7	91.4	93.1
15×15	63.4	92.0	94.4
20×20	58.8	90.1	94.3

(b).			
	*Angular Correction*		
*Field Size (cm* ^*2*^ *)*	*No Correction*	*Central Correction*	*Entire Correction*
10×10	64.3	91.7	93.0
15×15	60.3	93.3	94.6
20×20	56.7	93.8	95.6

### C. Dose verifications of various simple plans

**Figure 8:**
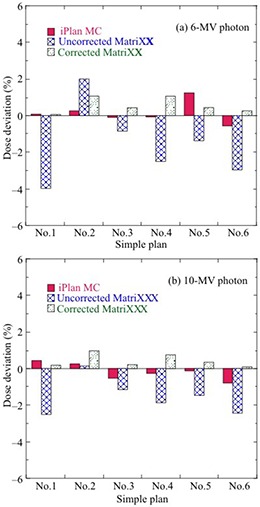
Deviations of iPlan MC‐calculated and MatriXX‐measured doses without and with angular correction from PTW‐TN31010 doses. Dose verifications were performed for various simple plans (Table 1) with a $10\times 10{\;\text{cm}}^{2}$ field for (a) 6 MV and (b) 10 MV photons.

Figure 8 shows deviations of iPlan MC‐calculated and MatriXX‐measured doses without and with the angular correction from PTW‐TN31010 doses. The dose verifications were performed for the simple plans presented in Table 1. The MC‐calculated and corrected MatriXX doses were within 1.2% of the PTW‐TN31010 doses in all plans for 6 MV and 10 MV photons. In contrast, the maximum dose deviations of the uncorrected MatriXX were ‐4% and −2.5% in simple plan 1 for 6 MV and 10 MV photons, respectively. The results of the gamma evaluation between iPlan MC‐calculated and MatriXX dose distributions without and with “central correction” or “entire correction” for various simple plans in a $10\times 10{\;\text{cm}}^{2}$ field of 6 MV photons are presented in Table 5. For simple plan 1 at $\theta =0^{{}^{\circ}}$ and 180°, the passing rates of corrected MatriXX dose distributions were improved by 33.3% and 27.9% compared to 57.4% and 71.8% without the correction at the 2%/2 mm and 3%/3 mm criteria, respectively. Similarly, for simple plan 2 at θ=90° and 270°, the passing rates of MatriXX dose distributions with “central correction” decreased by 8.7% and 1.5% compared to 90.0% and 99.3% without the correction at the 2%/2 mm and 3%/3 mm criteria, respectively. In contrast, the passing rates of MatriXX dose distributions with “entire correction” were improved by 9.4% and 1.8% compared to 81.3% and 97.8% with “central correction” at the 2%/2 mm and 3%/3 mm criteria, respectively. In all other plans, the passing rates for the “central” and “entire” corrections were almost identical. The results for other fields were also similar to those for the $10\times 10{\;\text{cm}}^{2}$ field.

**Table 5:** Comparison in passing rates (%) between iPlan MC‐calculated and MatriXX dose distributions without and with “central correction” or “entire correction” for various simple plans in a $10\times 10\;{\text{cm}}^{2}$ field of 6 MV photons. Gamma evaluation was performed with 2% dose difference and 2 mm DTA, and 3% dose difference and 3 mm DTA.

	*Angular Correction*					
	*No Correction*		*Central Correction*		*Entire Correction*	
*Plan No*.	*2%/2 mm*	*3%/3 mm*	*2%/2 mm*	*3%/3 mm*	*2%/2 mm*	*3%/3 mm*
1	57.4	71.8	90.1	99.5	90.7	99.7
2	90.0	99.3	81.3	97.8	90.7	99.6
3	88.4	98.8	92.1	99.3	94.5	99.3
4	68.2	93.8	95.5	99.7	95.6	99.7
5	80.4	96.9	95.2	99.2	95.2	99.2
6	78.9	96.6	96.2	99.9	96.2	99.9

### D. Dose verifications of IMRT and VMAT plans

**Figure 9:**
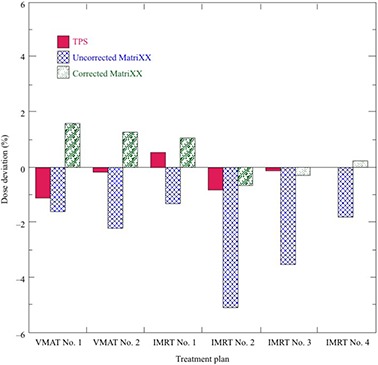
Deviations of TPS‐calculated and MatriXX‐measured doses with and without angular correction from PTW‐TN31010 doses. Dose verifications were performed with the IMRT (iPlan MC) and VMAT (Eclipse AAA) plans presented in Table 2.

Figure 9 shows the deviations of TPS‐calculated and MatriXX‐measured doses with and without angular correction from the PTW‐TN31010 doses. The dose verifications were performed for the IMRT (iPlan MC) and VMAT (Eclipse AAA) plans presented in Table 2. The TPS‐calculated doses had a median deviation of −0.2% with a range of −1.1% to +0.5% relative to the PTW‐TN31010 doses. Similarly, the uncorrected MatriXX doses had a median deviation of −2.0% with a range of −5.1% to −1.3%. In contrast, MatriXX doses with “entire correction” had a median deviation of 0.6% with a range of −0.7% to +1.6%. The results in the gamma evaluation between the TPS‐calculated and MatriXX dose distributions without and with “central correction” or “entire correction” are presented in Table 6. For VMAT plan 1, passing rates of corrected MatriXX dose distributions were improved 0.8% and 0.9% compared to 90.7% and 97.7% without the correction at the 2%/2 mm and 3%/3 mm criteria, respectively. Similarly, passing rates of corrected MatriXX dose distributions for IMRT plan 5 were improved by 27.3% and 17.9% compared to 60.4% and 77.5% without the correction, respectively. The passing rates of MatriXX dose distribution with “entire correction” were only 0.8% improvement compared to 86.9% and 94.6% with “central correction” at the 2%/2 mm and 3%/3 mm criteria, respectively. The passing rates among RTQA2‐measured, TPS‐calculated, and MatriXX dose distributions with “entire correction” are presented in Table 7. The passing rates at 3% dose tolerance and 3 mm DTA were more than 94% for all plans.

**Table 6:** Comparison in passing rates (%) between TPS‐calculated and MatriXX dose distributions without and with “central correction” or “entire correction” for IMRT (iPlan MC) and VMAT (Eclipse AAA) plans. Gamma evaluation was performed with 2% dose difference and 2 mm DTA, and 3% dose difference and 3 mm DTA.

	*Angular Correction*					
	*No Correction*		*Central Correction*		*Entire Correction*	
*Plan No*.	*2%/2 mm*	*3%/3 mm*	*2%/2 mm*	*3%/3 mm*	*2%/2 mm*	*3%/3 mm*
1 (VMAT)	90.7	97.7	91.3	98.3	91.5	98.6
2 (VMAT)	92.9	99.1	93.4	99.1	93.9	99.1
3 (IMRT)	87.9	97.9	94.8	99.8	94.9	99.8
4 (IMRT)	65.8	87.2	87.0	99.2	87.1	99.2
5 (IMRT)	60.4	77.5	86.9	94.6	87.7	95.4
6 (IMRT)	82.8	99.2	95.1	99.6	95.6	99.6

**Table 7:** Comparison in passing rates (%) among RTQA2‐measured and TPS‐calculated dose distributions and MatriXX dose distributions with “entire correction” for IMRT (iPlan MC) and VMAT (Eclipse AAA) plans. Gamma evaluation was performed with 3% dose difference and 3 mm DTA.

*Plan No*.	*RTQA2 and TPS*	*RTQA2 and MatriXX*	*MatriXX and TPS*
1 (VMAT)	95.7	96.1	98.6
2 (VMAT)	98.5	94.6	99.1
3 (IMRT)	96.0	95.4	99.8
4 (IMRT)	96.0	95.8	99.2
5 (IMRT)	97.3	95.5	95.4
6 (IMRT)	96.6	94.8	99.6

## IV. DISCUSSION

Dosimetric characteristics such as detector stability, dose linearity, dose rate, and energy dependence are required for the composite dose verification of IMRT and VMAT for 2D detector arrays.^11^, ^19^ In the current study, we focused on the angular dependence of 2D detector arrays, and evaluated this dependence for the central and off‐axis detectors of MatriXX. As shown in Fig. 3, the angular dependence of MatriXX depended on the photon energy and geometrical position of the detectors. The angular dependence can most probably be explained by the perturbation effect occurring at the interface between air and the high‐Z material. Our results were consistent with those reported by Wolfsberger et al.^(23)^ and Boggula et al.^(30)^ on the angular dependence of central and off‐axis detectors. This study applied off‐axis CFs for MatriXX detectors on the 16th and 17th rows, not all off‐axis CFs. With off‐axis CFs it is also assumed that the angular dependence is almost the same for the cross‐line and in‐line planes of MatriXX detectors. Boggula et al.^(30)^ showed slight difference in the angular dependence of MatriXX detectors for cross‐line and in‐line planes. In this study, the angular dependence for the cross‐line and in‐line planes of MatriXX detectors was compared with MatriXX‐measured and MC‐calculated distributions for field sizes of $10\times 10{\;\text{cm}}^{2}$, $15\times 15{\;\text{cm}}^{2}$, and $20\times 20{\;\text{cm}}^{2}$. As shown in Table 4, MatriXX‐measured distributions with the angular correction were high passing rates independent of field sizes for the strict gamma criteria of 2%/2 mm. Therefore, off‐axis CFs for MatriXX detectors used in this study are reasonable for the composite dose verification of IMRT and VMAT. In addition, since CFs for MatriXX are obtained by excluding the couch top, they are applicable to using the device on any other couch top.

The angular dependence of MatriXX was determined using the two correction methods (central and entire correction) and then validated under various fields ($10\times 10{\;\text{cm}}^{2}$, $15\times 15{\;\text{cm}}^{2}$, and $20\times 20{\;\text{cm}}^{2}$) and simple plans. As shown in Figs. 4 and 5, uncorrected MatriXX doses underestimated the doses measured with the ionization chamber. MatriXX doses with “central correction” agreed well with ionization chamber‐measured doses at the central axis; however, they showed discrepancies of up to 3.9% and 3.0% for 6 MV and 10 MV photons, respectively, at off‐axis locations. This was caused by the use of the CF of central detectors for off‐axis detectors. In contrast, MatriXX doses with “entire correction” agreed well with doses measured with the ionization chamber for both central and off‐axis detectors. As shown in Fig. 6 and Table 5, the differences in the passing rates between “central correction” and “entire correction” were up to 14% for each beam with a $10\times 10{\;\text{cm}}^{2}$ field, 9.4% for plans with a number of lateral beams, and within 2% for plans with multiple gantry angles. This was because of the dose compensation by irradiation from multiple gantry angles. However, the accuracy of MatriXX measurement was improved using “entire correction” in simple plans with a number of lateral beams for a 6 MV photon.

MatriXX dosimetry with angular correction was validated by measurements with the ionization chamber and RTQA2 for the composite dose verifications of IMRT and VMAT plans. As shown in Fig. 9 and Tables 6 and 7, MatriXX dosimetry with angular correction agreed well with the ionization chamber, RTQA2, and TPS for both absolute doses and dose distributions. Wolfsberger et al.^(23)^ reported that a dose bias of up to ‐3% can be observed for dose verifications of IMRT and VMAT plans if not corrected for the angular dependence of MatriXX. In this study, a dose bias of up to −5.1% was observed for dose verifications of IMRT plans. However, this was improved to within 2% by correcting the angular dependence of MatriXX. Recently, Kruse^(41)^ reported on single planar dosimetry for IMRT QA and concluded that effective patient‐specific IMRT QA should still include composite dose measurements in the complete plan. Although MatriXX was originally designed as a single planar dosimeter, it can be used by considering the angular dependence for the composite dose verification of IMRT plans. O'Daniel et al.^(29)^ verified 39 VMAT plans by ionization chamber, film, and MatriXX measurements without the angular correction. They reported that ionization chamber and MatriXX measurements gave very similar results; however, discrepancies of up to 3% were observed in certain cases. These discrepancies are most likely due to the angular dependence of MatriXX. Our results for MatriXX agreed to within 2% of the ionization measurements for IMRT and VMAT treatment plans when using the “entire correction” method. For the QA plans in this study, the effect of angular correction for MatriXX dosimetry was observed in IMRT QA but not in VMAT QA. Boggula et al.^(30)^ investigated the suitability of MatriXX for VMAT QA and reported an improvement of 4.3% for gamma criteria of 2%/ 2 mm by applying the CFs. Moreover, when the gamma criteria were relaxed to 3%/ 3 mm, the passing rate was more than 95% and approximately 100% without and with corrections, respectively. In this study, the passing rates for IMRT QA and VMAT QA were improved up to 27.3% and 1% at 2%/ 2 mm, respectively. The angular dependence of MatriXX for VMAT QA may be compensated by irradiation from multiple gantry angles. The differences in an improvement of the passing rates between our results and Boggula et al.^(30)^ for VMAT QA are due to a difference in the number of arcs.

The results of the current study showed that the accuracy of dose measurement with MatriXX is improved by correcting the angular dependence. However, another problem of MatriXX is its spatial resolution. Poppe et al.^(16)^ and Herzen et al.^(17)^ suggested a convolution correction method that considers the response function of each detector. This method might enhance the verification results for dose distribution. Moreover, Schreibmann et al.^(21)^ and O'Daniel et al.^(29)^ reported that MatriXX produces a lower passing rate for small fields and fields with a high‐dose gradient in the center of the treatment volume. For such cases, MatriXX measurements should be validated with film measurements.

## V. CONCLUSIONS

This study evaluated the angular dependence of central and off‐axis detectors of MatriXX. The angular dependence magnitudes of the central detectors differed by up to 7% from those of the off‐axis detectors. To resolve this problem, we established a correction method for both these detectors. The accuracy of dose measurement in MatriXX with our correction method was improved for both absolute doses and dose distributions. In particular, the correction method showed improvements for a treatment plan with a number of lateral beams for low‐energy photons. MatriXX with angular correction was useful for the composite dose verification of IMRT or VMAT plans.

## ACKNOWLEDGMENTS

The authors would like to thank Noriyuki Araki and Shunji Saiga of Toyo Medic Company in Japan for support in making the special phantom for the PTW‐TN31010 chamber measurements. The authors also would like to thank the reviewers and editors for their helpful comments.
